# Construction and Verification of Immunohistochemistry Parameters-Based Classifier to Predict Local-Recurrence of Upper Tract Urothelial Carcinoma After Kidney-Sparing Surgery

**DOI:** 10.3389/fonc.2022.872432

**Published:** 2022-05-04

**Authors:** Xu Cheng, Wentao Liu, Yijian Li, Yinhuai Wang

**Affiliations:** Department of Urology, The Second Xiangya Hospital of Central South University, Changsha, China

**Keywords:** kidney-sparing surgery, upper tract urothelial carcinoma, recurrence, nomogram, intravesical chemotherapy

## Abstract

**Background:**

Kidney-sparing surgery (KSS) for upper tract urothelial carcinomas (UTUCs) has been gradually performed in selected patients beyond the recommendation of guidelines. However, there is still a lack of tools to evaluate postoperative local recurrence. Herein, a new nomogram was established to predict the local recurrence risk after KSS.

**Methods:**

Patients were randomly divided into two cohorts (training: testing cohorts = 7:3). Cancer samples after KSS were used for immunohistochemical tests to detect molecules missing in previous pathology reports. Then, the total number of molecules were screened by the least absolute shrinkage and selection operator (LASSO) method to construct an IHCscore, which was further tested in the validation cohort. Finally, the IHCscore and other clinicopathologic parameters were combined to develop a more accurate model using univariate and multivariate Cox regression methods.

**Results:**

In total, 200 patients were included. The Kaplan–Meier test showed that high Ki-67 and loss of Uroplakin III and E-cadherin were correlated with poor recurrence-free survival. The individual IHCscore was calculated based on the expression levels of Ki-67, Her2 and E-cadherin. Based on the IHC score, patients were further classified as low- or high-risk, and a significant difference in the recurrence-free survival was observed between the two groups. Then, the nomogram was developed based on Gender, surgical margin and IHCscore; this nomogram had a higher AUC (0.847) in predicting 3-year recurrence-free survival than the IHCscore alone (0.788).

**Conclusions:**

This easy-to-use nomogram shows better prediction accuracy in recurrence-free survival after KSS and may guide individualized intravesical chemotherapy. However, a larger sample is required for external validation.

## Introduction

Upper tract urothelial carcinoma (UTUC) is an uncommon tumor and only comprises 5–10% of urothelial cell carcinomas, but the incidence has an annually increasing trend worldwide (1). Due to its rarity, high-quality evidence regarding risk stratification is lacking to guide treatment. Radical nephroureterectomy (RNU) is the gold standard treatment for localized UTUC, with a postoperative recurrence rate of 22–47% in the bladder (2) and 2–6% in the contralateral upper tract (3), which greatly increases the weight of the disease burden for patients. It is well accepted that patient-specific factors, tumor-specific factors, and treatment-specific factors are significant predictors of bladder recurrence after RNU (4), while emerging studies have focused on the molecular aspects of the disease, which may lead to a better understanding of UTUC. Molecular characterizations of UTUC, such as Ki-67, Her2, P16, P53, Uroplakin III, Microsatellite instability (MSI), Snail, E-Cadherin and PD-L1, have been shown to be associated with tumor behavior and oncological outcomes (5–10). However, there is still a lack of studies combining these molecules for prognostic evaluation in UTUC.

Kidney-sparing surgery (KSS) for low-risk UTUC can preserve more renal function without compromising oncological outcomes (11) and is gaining acceptance for selected high-risk patients beyond the recommendation of current guidelines. High-level evidence comparing the recurrence rate between KSS and RNU is scant and hard to find, but sufficient studies have demonstrated that intravesical chemotherapy could effectively reduce the bladder recurrence rate after RNU (2, 12). However, the current selection of agents and treatment cycle remain controversial.

Herein, we comprehensively evaluated the molecular characterization in UTUC and developed a molecules-based immunohistochemistry (IHC) score (IHCscore) to predict the recurrence-free survival of patients with UTUC receiving KSS. Moreover, the IHCscore was integrated with clinicopathologic characteristics to construct a nomogram of the prognostic model, which may guide more efficient and individualized intravesical chemotherapy strategies in these patients.

## Methods

### Study Design and Participants

**Figure 1:**
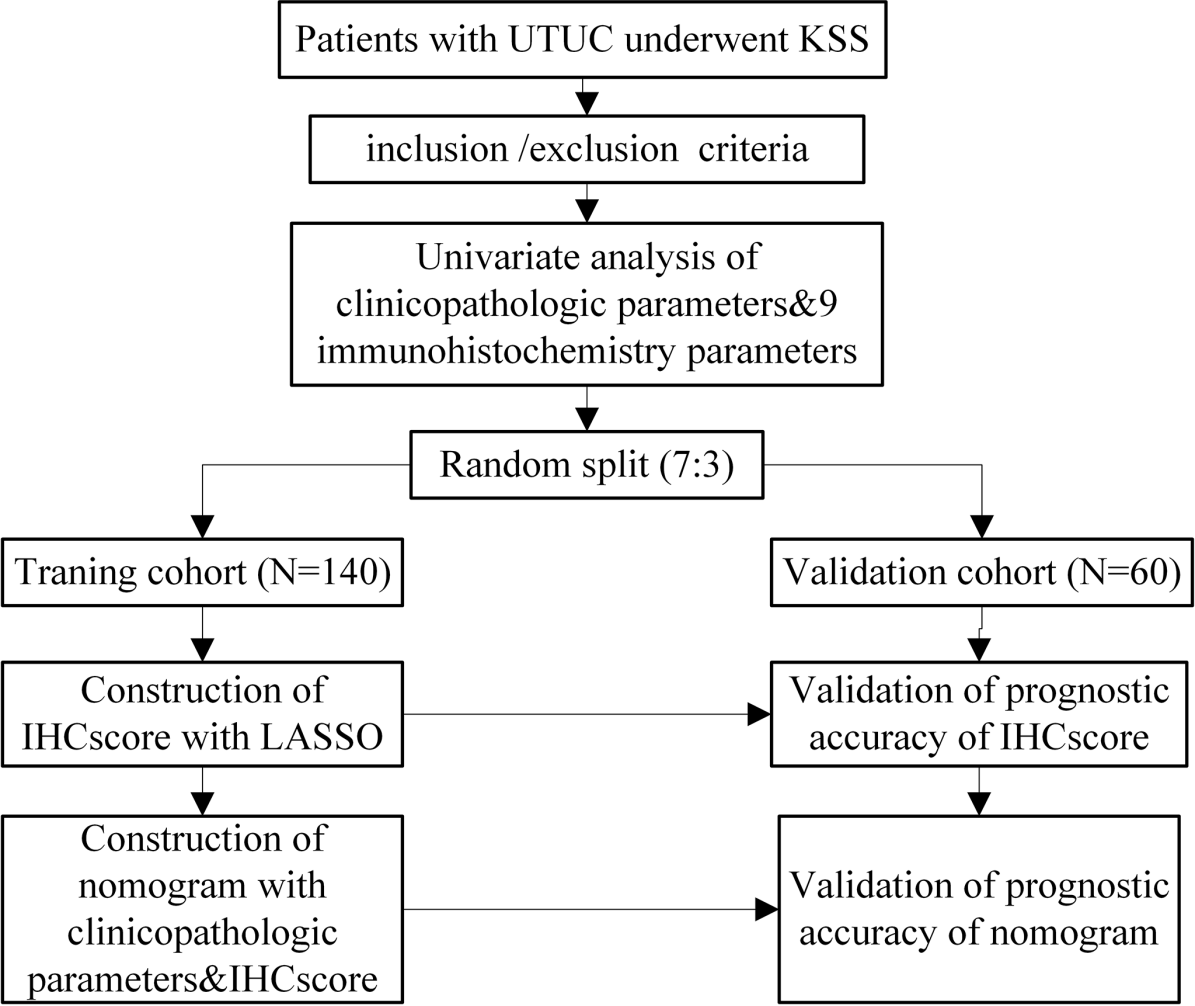
A flow-diagram of the present study.

The study design is shown in **Figure 1**. Patients with UTUC at Xiangya Second Affiliated Hospital, Central South University from November 1st, 2012, to June 1st, 2021, were retrospectively enrolled in the study. The present study was approved by the Ethics Committee of the Second Xiangya Hospital, Central South University. The inclusion criteria of this study were as follows: (1) patients aged 18 years and older who were pathologically diagnosed with UTUC and underwent KSS in our center; (2) patients with complete clinical records for the present study. The exclusion criteria were as follows: (1) patients diagnosed with other occurrent malignant tumors; (2) patients who were lost to follow-up; and (3) patients with missing or unscorable pathological sections. All the included patients were randomly categorized into training and validation cohorts at a ratio of 7:3.

### Data Collection

The candidate variables shown in **Table 1**, including patient-specific factors, tumor-specific factors, treatment-specific factors and molecular factors, were selected based on published studies (4–10), accessibility, and professional knowledge. These data were retrospectively collected from the medical records. For missing data of the four molecules (Her2, P16, E-cadherin, and Snail) in previous immunohistochemistry reports, paraffin-embedded tumor sections were obtained for immunohistochemistry again and were evaluated by a pathologist from the center. Tumor stage and grade were evaluated according to the Tumor Node Metastasis classification (2017 version) and the World Health Organization classification (2016 version).

**Table 1:** Characteristics of patients with UTUC after kidney-sparing surgery.

Variables	Total (n = 200)	Training cohort (n = 140)	Validation cohort (n = 60)		*t/χ^2^*	P Value
**Age (year)**	65.38 (9.190)	65.77 (9.210)		64.47 (9.155)	0.920	0.359
**Gender**					1.194	0.349
Female		63		22		
Male		77		38		
**BMI (kg/m^2^)**	22.06 (3.450)	22.07 (3.502)		22.03 (3.355)	0.080	0.936
**ASA grade**					0.397	0.637
>II	120	86		34		
≤II	80	54		26		
**Smoke, N (%)**	36 (18)	25 (17.85)		11 (18.33)	0.006	1.000
**^a^Comorbidity**						0.401
<2	184	127		57		
≥2	16	13		3		
**Previous BCa, N (%)**	13 (6.5)	10 (7.14)		3 (5)		0.758
**Hydronephrosis, N (%)**	166 (83)	144 (81.43)		52 (86.67)	0.817	0.418
**Tumor size (cm)**	2.84 (1.68)	2.84 (1.689)		2.85 (1.671)	-0.490	0.961
**Distal ureter tumor, N (%)**	121 (60.5)	82 (58.57)		39 (65)	0.726	0.433
**pT stage**					0.971	0.351
T≤2	158	108		50		
T>2	42	32		10		
**High grade, N (%)**	141 (70.5)	95 (67.86)		46 (76.67)	1.561	0.239
**Positive margin, N (%)**	21 (10.5)	13 (9.29)		8 (13.33)	0.732	0.452
**Lymph node invasion, N (%)**	13 (6.5)	8 (5.71)		5 (8.33)		0.536
**Surgical approach**					1.69	0.208
SRA	77	58		19		
UR	123	82		41		
**Lymph node dissection, N (%)**	24 (12)	16 (11.43)		8 (13.33)	0.144	0.813
**Ki-67, N (%)**	22.31 (13.251)	22.94 (13.987)		20.85 (11.322)	1.024	0.307
**Positive her2, N (%)**	37 (18.5)	29 (20.71)		8 (13.33)	1.518	0.240
**Positive p16, N (%)**	96 (48)	69 (49.29)		27 (45)	0.309	0.644
**Positive p53, N (%)**	65 (32.5)	42 (30)		23 (38.33)	1.330	0.323
**Positive uroplakin III, N (%)**	95 (47.5)	66 (47.14)		29 (48.33)	0.024	1.000
**^b^MSI**					0.061	1.000
stable	133	93		40		
low	22	15		7		
high	45	32		13		
**Positive snail (%)**	76 (38)	49 (35)		27 (45)	1.783	0.205
**Positive E-Cadherin, N (%)**	95 (47.5)	62 (44.29)		33 (55)	1.933	0.216
**PD-L1, (CPS score)**	7.26 (7.009)	7.35 (7.081)		7.05 (6.892)	0.277	0.782

BCa, bladder cancer; SRA, Segmental resection and anastomosis; UR,Ureterectomy and replantation; ^a^Number of comorbidities including diabetes, coronary heart disease, hypertension, hyperlipidemia; MSI, Microsatellite instability, ^b^Loss of expression of MLH1、MSH2、MSH6 and PMS2 was defined as MSI (only one loss, low, two or more than two loss, high); CPS, Combined Positive Score.

In this study, recurrence was screened by computerized tomography or cystoscopy during the follow-up, and tumors occurring in the upper tract or bladder were considered recurrence. The end point was recurrence-free survival (RFS), defined as the time from the KSS to 1) recurrence and 2) all-cause death. In addition, the number of patients lost to follow-up was calculated; when more than 10% of patients were lost to follow-up, their baseline characteristics were further evaluated.

### Immunohistochemistry

The immunohistochemical procedures were performed following the previous literature (13). The paraffin-embedded tissue sections were incubated with the following antibodies: anti-Her2 (cat. ab134182, dilution 1:200), anti-P16 (cat. ab151303, dilution 1:500), anti-E-cadherin (cat. ab40772, dilution 1:500), and anti-Snail (cat. ab180714, dilution 1:200), which was obtained from Abcam (Cambridge, UK). The prepared slides were then observed under a microscope for target protein detection.

### Statistical Analysis

The measurement data are presented as the mean with standard deviation or median with 95% confidence interval, and the count data are presented in the form of a number (percentage). For two-group comparison, two unpaired sample t-test was used for measurement data, and chi-square tests or Fisher’s exact tests were used for count data. The Kaplan–Meier method with a log-rank test was used for survival analysis. The reverse Kaplan–Meier method was used to calculate the median follow-up period. By using the ‘glmnet’ R package, we selected candidate molecular variables with the least absolute shrinkage and selection operator (LASSO) Cox regression method, and the generated coefficients were adopted to construct an IHCscore. Based on the follow-up data, we employed the enumeration method to generate an optimal cutoff value of the IHCscore and divided the patients into high- and low-risk groups. We then performed a univariate & multivariate analysis of the IHCscore and other clinicopathologic variables using the Cox regression method. The generated coefficients were adopted to develop a nomogram, and the ‘rms’ package of R was employed to draw calibration plots of the new model. The predictive value of the two new models over three years was assessed by time-dependent receiver operating characteristic (ROC) analysis with the ‘survivalROC’ package of R. To evaluate the clinical usefulness of the nomogram, we carried out decision curve analysis with the ‘stdca’ package of R. Statistical significance was considered when two-sided p<0.05. SPSS (version 22.0) and R software (Version: 4.1.2) were employed for all statistical analyses.

## Results

### Baseline and Clinical Characteristics of Patients with UTUC

**Figure 2:**
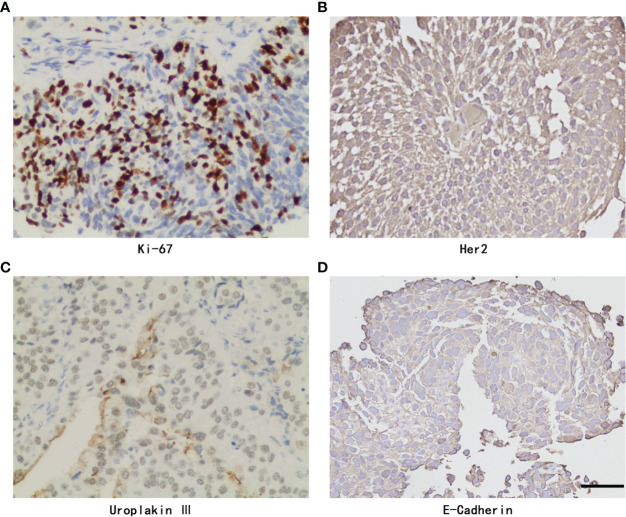
Representative Immunohistochemistry staining of Ki-67, Her2, Uroplakin III, E-Cadherin and PD-L1 with positive expression in UTUC. **(A)** Ki-67 staining. **(B)** Her2 staining. **(C)** Uroplakin III staining. **(D)** E-Cadherin staining. original magnifcation,×400. Scale bar, 50μm.

Eleven of 211 patients in the primary cohort were lost to follow-up and were excluded, which is less than 10%. The whole cohort (n=200) was divided into a training set (n=140) and validation set (n=60). **Table 1** presents a univariate analysis of all the clinicopathologic variables in the two cohorts. In the whole cohort (n=200), 71 (35.5%) patients developed local recurrence during follow-up. The median period of follow-up was 48.000 (44.473, 51.527) months. All the shown variables were not different between the two cohorts (p>0.05). For the IHC variables, the significant molecules (such as Ki-67, Her2, Uroplakin III and E-cadherin) in the following analysis are shown in **Figure 2**.

### Prognostic Values of the Nine Immunohistochemistry Parameters

**Figure 3:**
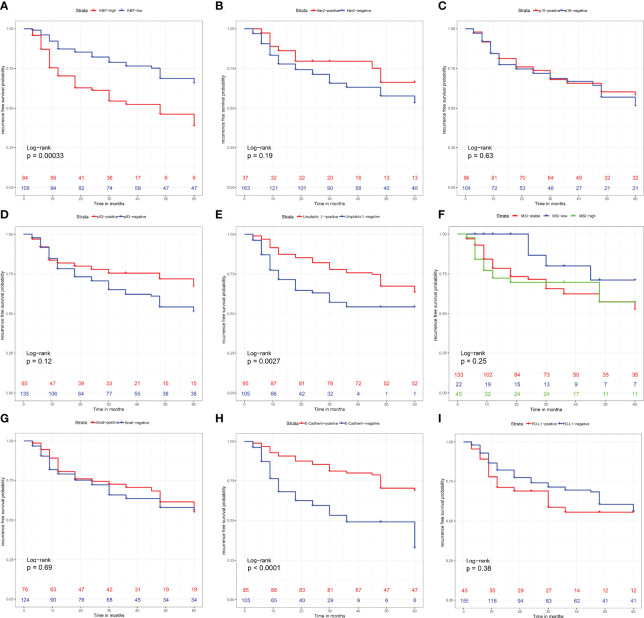
Recurrence-free survival analysis with Kaplan-Meier method for patients divided by **(A)** Ki-67 expression level, **(B)** Her2 expression level, **(C)** P16 expression level, **(D)** P53 expression level, **(E)** Uroplakin III expression level, **(F)** Microsatellite instability status, **(G)** Snail expression level, **(H)** E-Cadherin expression level and **(I)** PD-L1 expression level. P values were determined using the log-rank test.

Surviving curves showed that high Ki-67, loss of Uroplakin III and E-cadherin were related to worse RFS (P <0.05) (**Figure 3**). However, expression of P16, Snail, PD-L1, and MSI status were not relevant to the RFS of the UTUC patients (P >0.05) (**Figure 3**). Notably, the expression of Her2 and P53 showed borderline significance in RFS (P =0.19 and P =0.12, respectively).

### Establishment and Evaluation of the IHCscore

**Figure 4:**
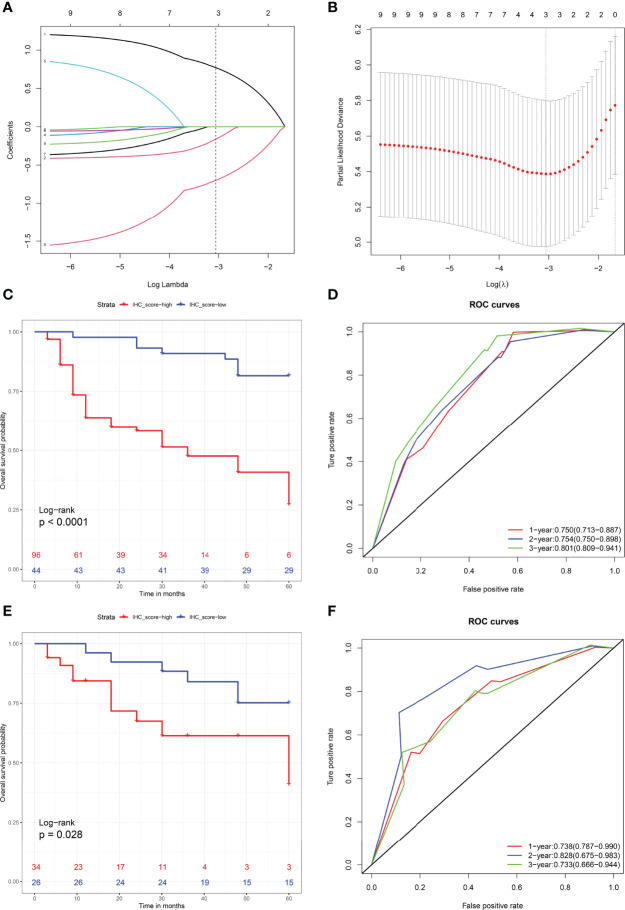
**(A)** LASSO coefcient profles of the nigh molecules. Number 1-9 respectively represent Ki-67, Her2, P16, P53, Uroplakin III, Microsatellite instability status, Snail, E-Cadherin and PD-L1. **(B)** Partial likelihood deviance for LASSO coefficient profiles by in 10-times cross-validation. Vertical black dotted line represent an optimal values tuning parameter (λ). **(C, D)** Recurrence-free survival analysis with Kaplan-Meier method for patients divided by IHCscore in the training **(C)** and validation sets **(D)**. **(E, F)** ROC with AUC of recurrence-free survival at 1-, 2-, and 3-year predicted by IHCscore in the training **(E)** and validation **(F)** datasets.

To further explore the value of the immunohistochemistry parameters in predicting RFS after KSS, the IHCscore for individual patients was calculated using the formula IHC score = 0.7709 × Ki-67 status - 0.1622 × Her2 status - 0.6997 × E-cadherin status, which was generated by the LASSO Cox model (**Figures 4A, B**). Patients in the training set were classified into low- and high-risk groups according to the best cutoff value of the IHC score found by the enumeration method. A higher IHC score was significantly correlated with a worse RFS (P < 0.001) (**Figure 4C**), and a worse RFS was consistently observed in patients with a high IHCscore in the validation cohort (P < 0.05) (**Figure 4D**). Then, we further evaluated the discrimination performance of the developed IHCscore by calculating the area under the curve (AUC) time-dependent ROC curve. The AUC at 1, 2 and 3 years suggested that the IHCscore had a good discrimination ability in both datasets (**Figures 4E, F**). Collectively, the IHCscore might not have a poor predictive ability and can be used as a supplemental means of predicting RFS after KSS.

### Development of a Nomogram Combining Potential Clinicopathologic Variables and IHCscore

**Figure 5:**
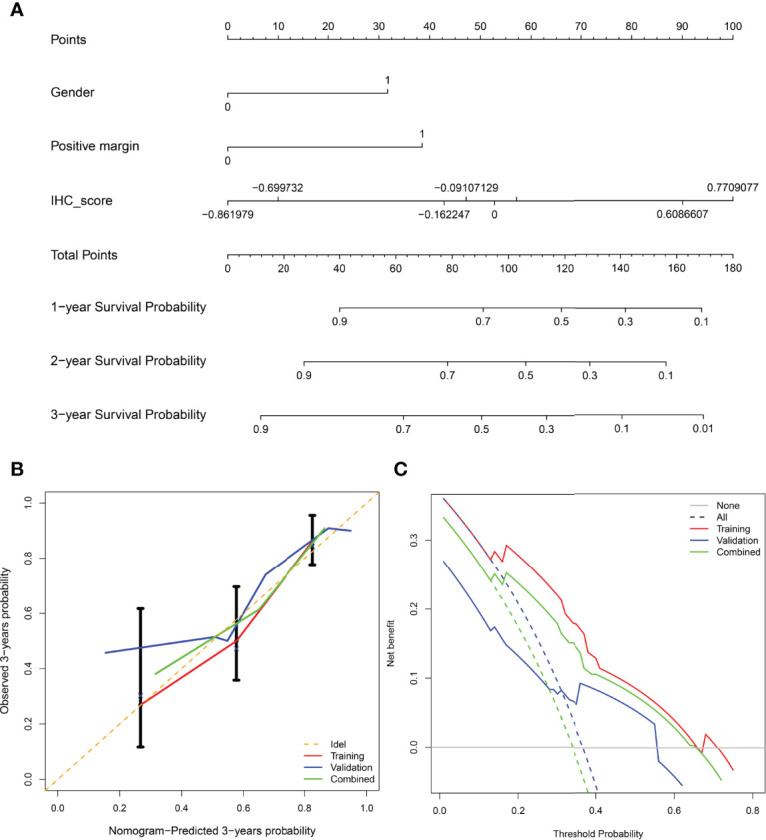
**(A)** Nomogram for predicting recurrence-free survival of patients at 1,2,3-year. **(B)** Calibration curve of the nomogram in predicting recurrence-free survival at 3-years in the training cohort, validation cohort, and combined cohort. X-axis: prediction of recurrence in patients. Y-axis: actual diagnosed recurrence.**(C)** Decision curve of the nomogram for 3-year recurrence-free survival in three cohorts.

**Figure 6:**
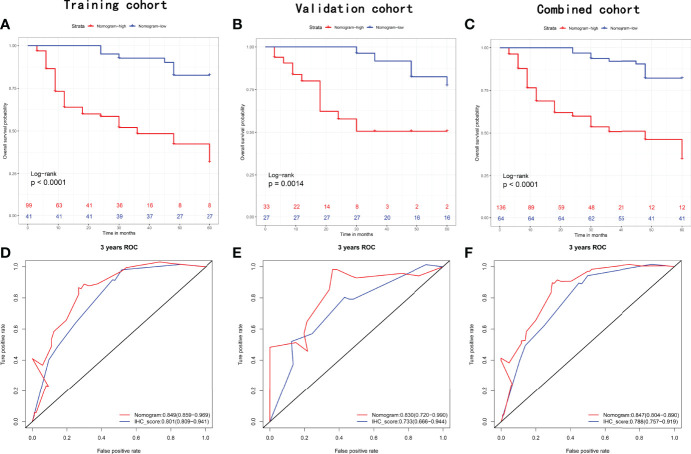
Recurrence-free survival analysis with Kaplan-Meier method for patients divided by nomogram in the **(A)** training set, **(B)** validation set, and **(C)** combined set. ROC with AUC of recurrence-free survival at 3-year predicted by nomogram and IHCscore in the **(D)** training set, **(E)** validation set, and **(F)** combined set.

Considering the potential prognostic value of other clinicopathologic variables, we combined these parameters with the established IHCscore to develop a better model to predict RFS after KSS. After the univariate & multivariate analysis, gender, surgical margin and IHCscore were finally selected to independently predict RFS in the training cohort (**Supplementary Table 1**). Then, a nomogram was constructed based on the three parameters to predict the RFS at 1, 2, and 3 years (**Figure 5A**). The calibration curves for the probability of 3-year RFS in the training cohort (C-index 0.769 (0.711, 0.826)), the validation cohort (C-index 0.781 (0.680, 0.882)), and the combined cohort (C-index (0.723, 0.819)) showed favorable consistency between the nomogram prediction and the actual observation (**Figure 5B**). Net benefits were evaluated by decision curve analysis in the two datasets, and the satisfactory positive net benefits at the threshold probabilities suggested that the nomogram had good clinical usefulness for 3-year RFS prediction (**Figure 5C**). In addition, the patients classified as low risk of recurrence by the nomogram had better RFS than high-risk patients in the three datasets (**Figures 6A–C**). The nomogram and the simple IHC score were further compared by ROC analysis. A better AUC of the nomogram was shown in the three datasets (**Figures 6D–F**).

## Discussion

A nomogram is a visible way to show the regression results, which can express the significance of different variables on the outcome by the length of the line. By calculating the total score, the probability of the outcome in the patient can be visually displayed. Some prognostic models before RNU have been established for locally advanced/muscle-invasive UTUC (14–17), and such nomograms originating from clinicopathologic variables are also available (17–24). However, owing to the uncommon UTUC case, these models are mostly based on retrospective studies or small cohorts. More importantly, the previous models mainly predict cancer-specific survival and overall survival after KNU, and none of them focus on local recurrence after KSS. Owing to a similarity of pathological characteristics between UTUC and bladder cancer, the diagnostic and treatment strategies for UTUC originated from studies on bladder cancer to some degree (25). However, molecular data now suggest that UTUCs and bladder cancer represent two distinct disease entities requiring different clinical management strategies. Recent molecular investigations have dramatically changed the perception of urothelial carcinomas, and the molecular features of UTUC might be highly related to clinicopathological characteristics as well as tumor outcomes (25, 26). At present, there is still no tool that includes these molecules for predicting the prognosis of UTUC. In our study, we comprehensively evaluated the molecular characterization in UTUC and developed a Ki67/Her2/E-cadherin-based IHCscore to predict the recurrence-free survival of patients with UTUC receiving KSS. Moreover, we combined the IHCscore with clinicopathologic variables and constructed a nomogram with a higher 3-year AUC in predicting RFS after KSS, which may be applied to guide more individualized intravesical chemotherapy strategies in these patients.

Nine risk factors were extracted from the literature review (5–10), considering collinearity, and following refinement by Lasso, only Ki67, Her2 and E-cadherin remained in Lasso regression to construct the IHCscore. Ki-67, a nuclear protein, is correlated with cellular proliferation, histological grade of the tumor and patient prognosis (27). Her2, one of the Epidermal Growth Factor Receptor protein family members, is an oncogenic receptor tyrosine kinase, and its upregulation was confirmed to be an aggressive behavior-related in many cancers, including urothelial carcinoma (28). E-cadherin, the binding protein between cells, plays a key role in neighboring cell adhesion, and loss of E-cadherin might be correlated with the invasion and metastasis of some epithelial cancers (29). Of note, variables included in the LASSO regression were not entirely consistent with the significant variables from univariate survival analysis (**Figures 3**, **4**). LASSO applied the penalized regression method to perform optimal variable combination selection and estimate the coefficients of the selected variables. Although Uroplakin III was significant according to the Kaplan–Meier test, the efficacy of Uroplakin III was too low to fit into the LASSO regression model.

The final nomogram included gender, positive margin and IHCscore and achieved a higher AUC than single IHCscore in predicting 3-year RFS (**Figures 6D–F**). A systematic review of previous literature has revealed several risk factors for bladder recurrence after RNU; among them, male sex and positive margins are risk factors, which supports the present nomogram to some extent (30). It is still noteworthy that tumor-related factors were replaced by IHCscore in the nomogram, and the nomogram still obtained good prediction accuracy. Interestingly, univariate Cox regression analysis identified that male gender, ASA>II, previous bladder cancer, hydronephrosis, pT stage>2, and positive margin were risk factors for recurrence after KSS (P<0.2, **Supplementary Table 1**), which is largely inconsistent with risk factors for cancer mortality, such as age, previous bladder cancer, smoking, tumor location, chronic kidney disease, clinical stage, tumor grade, lymphovascular invasion, multifocality, tumor diameter, invasive pT stage, etc. (14–24). This finding suggested that local recurrence does not always mean poor cancer-related survival.

Our data showed that patients receiving intravesical chemotherapy after KSS had a better RFS (P <0.0001) (**Figure S1**). The field cancerization hypothesis and intraluminal seeding are two hypotheses proposed to better explain bladder recurrence after KNU in UTUC (31). Based on two high-level systematic reviews of the literature, post-KNU immediate perfusion chemotherapy significantly decreases the risk of bladder recurrence of nonmetastatic UTUC (2, 12). Of note, the plausibility of the single-dose strategy is mainly based on intraluminal seeding theory. However, the recurrence risk might not be significantly reduced according to the field cancerization theory. Furthermore, a retrospective two-center study reported that a multiple instillation regimen better prevents bladder recurrence after KNU than single-dose instillation (32). Considering the few studies on KSS, the optimal post-KSS intravesical chemotherapy regimen to prevent local recurrence is far from conclusive. The nomogram may help to stratify patients into different risks of local recurrence and guide a more individualized intravesical chemotherapy regimen.

Indeed, our study still has some limitations. First, this is a retrospective, observational single-center retrospective study with a limited sample size, which easily produces sampling error and needs to be verified by data from larger samples. Second, because we used a nomogram, it is still necessary to determine which kind of intravesical chemotherapy regimen after KSS should be taken to maximize the benefits. Nonetheless, we can conclude that the developed model has good prediction accuracy in predicting RFS after KSS and is superior to the IHC score in our clinic. This easy-to-use nomogram may help with rapid clinical decision-making for intravesical chemotherapy regimens after KSS. However, a larger sample is required for external validation.

## Data Availability Statement

The raw data supporting the conclusions of this article will be made available by the authors, without undue reservation.

## Ethics Statement

The studies involving human participants were reviewed and approved by Ethics Committee of the Second Xiangya Hospital, Central South University. Written informed consent for participation was not required for this study in accordance with the national legislation and the institutional requirements.

## Author Contributions

Conception and design: YW, XC. Administrative support: YW, YL. Technical, or material support: XC, WL. Collection and assembly of data: XC, WL. Data analysis and interpretation: XC, WL, YL. Manuscript writing: XC. All authors contributed to the article and approved the submitted version.

## Funding

Funds received for open access publication fees from the Second Xiangya Hospital, Central South University.

## Conflict of Interest

The authors declare that the research was conducted in the absence of any commercial or financial relationships that could be construed as a potential conflict of interest.

## Publisher’s Note

All claims expressed in this article are solely those of the authors and do not necessarily represent those of their affiliated organizations, or those of the publisher, the editors and the reviewers. Any product that may be evaluated in this article, or claim that may be made by its manufacturer, is not guaranteed or endorsed by the publisher.

## Supplementary Material

Supplementary Figure 1 Recurrence-free survival analysis with Kaplan-Meier method for patients stratifed by intravesical chemotherapy or not.
