# Supplementary figures and images for: Construction and Verification of Immunohistochemistry Parameters-Based Classifier to Predict Local-Recurrence of Upper Tract Urothelial Carcinoma After Kidney-Sparing Surgery
Source: Front Oncol. 2022 May 4;12:872432. doi: 10.3389/fonc.2022.872432 (PMC9114713; doi:10.3389/fonc.2022.872432)

Strata intravesical instillation–Yes intravesical instillation–No

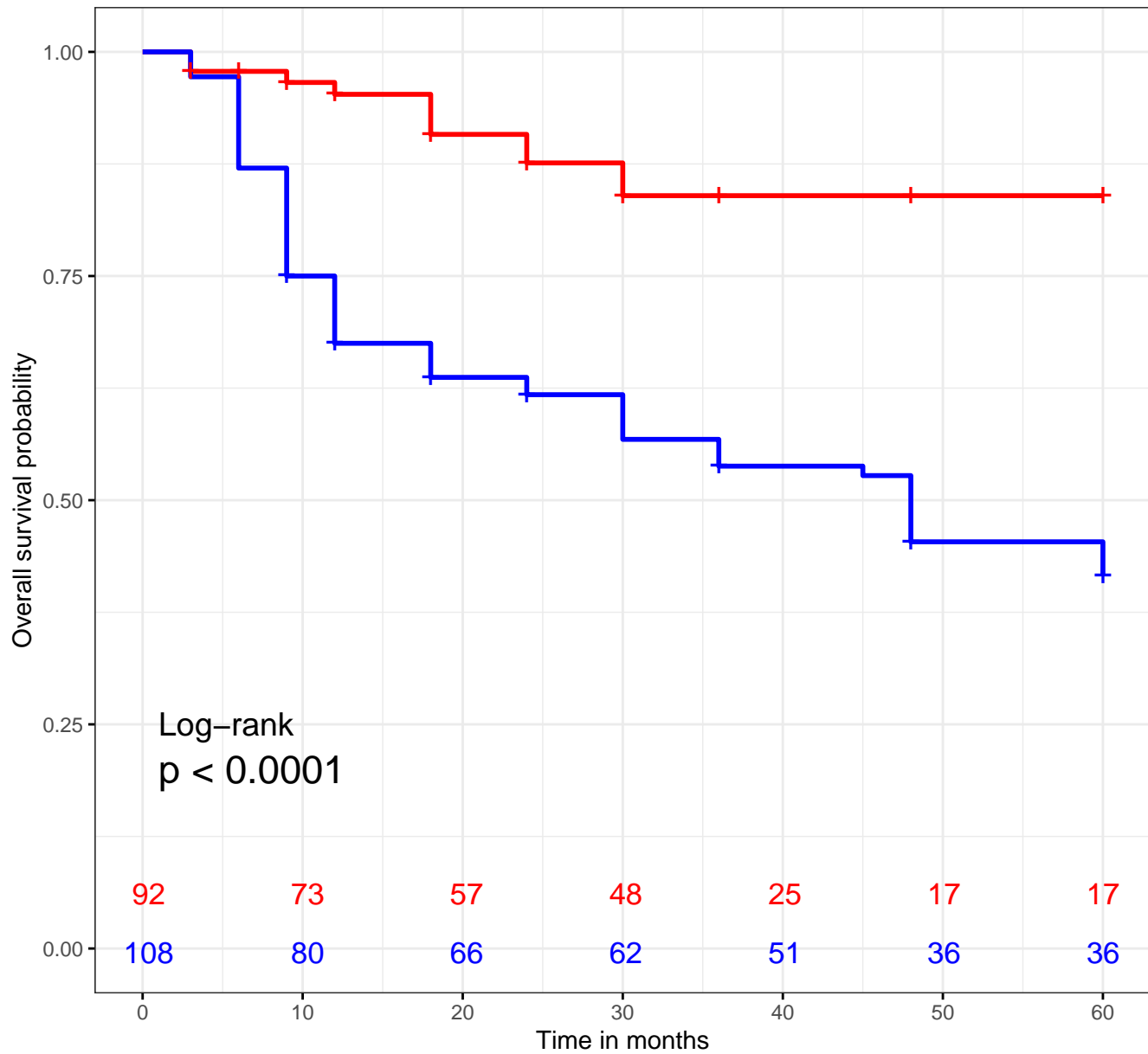

Supplement: Supplementary Figure 1 — Recurrence-free survival analysis with Kaplan-Meier method for patients stratifed by intravesical chemotherapy or not. [file DataSheet_1.pdf]
