# Supplementary material for: Construction and Verification of Immunohistochemistry Parameters-Based Classifier to Predict Local-Recurrence of Upper Tract Urothelial Carcinoma After Kidney-Sparing Surgery
Source: Front Oncol. 2022 May 4;12:872432. doi: 10.3389/fonc.2022.872432 (PMC9114713; doi:10.3389/fonc.2022.872432)
Supplement: Supplementary file 2 [file Table_1.doc]

Table 1. univariate & multivariate Cox analysis of the IHCscore and clinicopathologic parameters

|  | univariate Cox |  | multivariate Cox | | | |
| --- | --- | --- | --- | --- | --- | --- |
|  | P value |  | Coef | S.E. | Wald Z | Pr(>Z) |
| Age>=65 | 0.062 |  |  |  |  |  |
| Gender | 0.025 |  | 0.7592 | 0.2909 | 2.61 | 0.0091 |
| BMI>=24 | 0.578 |  |  |  |  |  |
| ASA>Ⅱ | 0.026 |  |  |  |  |  |
| Smoke | 0.216 |  |  |  |  |  |
| comorbidity | 0.461 |  |  |  |  |  |
| previous BCa | 0.153 |  |  |  |  |  |
| hydronephrosis | 0.106 |  |  |  |  |  |
| tumor size>3 | 0.449 |  |  |  |  |  |
| distal tumor location | 0.580 |  |  |  |  |  |
| pT stage>2 | 0.168 |  |  |  |  |  |
| High grade | 0.906 |  |  |  |  |  |
| Positive margin | 0.002 |  | 0.9224 | 0.3898 | 2.37 | 0.0180 |
| Lymph_node_invasion | 0.537 |  |  |  |  |  |
| surgical approach | 0.624 |  |  |  |  |  |
| lymphnode dissection | 0.885 |  |  |  |  |  |
| IHCscore | <0.0001 |  | 1.4662 | 0.2733 | 5.37 | <0.0001 |
